# Acute phase protein expressions in secretory and cistern lining epithelium tissues of the dairy cattle mammary gland during chronic mastitis caused by staphylococci

**DOI:** 10.1186/s12917-020-02544-8

**Published:** 2020-08-31

**Authors:** M. Zalewska, E. Kawecka-Grochocka, D. Słoniewska, E. Kościuczuk, S. Marczak, W. Jarmuż, L. Zwierzchowski, E. Bagnicka

**Affiliations:** 1 Institute of Microbiology, Faculty of Biology, University of Warsaw, Miecznikowa St. 1, Warsaw, Poland; 2 Department of Preclinical Sciences, Faculty of Veterinary Medicine, Warsaw University of Life Sciences SGGW, Nowoursynowska St, 166 Warsaw, Poland; 3 Department of Biotechnology and Nutrigenomics, Institute of Genetics and Animal Biotechnology PAS, Postępu 36A St., Jastrzębiec, Poland; 4 Present address: Robert H. Lurie Comprehensive Cancer Center, Northwestern University, 675 N St Clair St 21st Fl, Chicago, IL60611 USA; 5 Experimental Farm, Institute of Genetics and Animal Biotechnology PAS, Postępu 36A St., Jastrzębiec, Poland; 6 Department of Molecular Biology, Institute of Genetics and Animal Biotechnology PAS, Postępu 36A St., Jastrzębiec, Poland

**Keywords:** Dairy cow, Mammary gland, Epithelial cells, Cisternal lining epithelial cells, Chronic mastitis

## Abstract

**Background:**

Mastitis is the most common disease in dairy cattle and the costliest for the dairy farming industry, as it lowers milk yield and quality. Mastitis occurs as a result of interactions between microorganisms and the individual genetic predispositions of each animal. Thus, it is important to fully understand the mechanisms underlying these interactions. Elucidating the immune response mechanisms can determine which genetic background makes an animal highly resistant to mastitis. We analyzed the innate immune responses of dairy cows naturally infected with coagulase-positive staphylococci (CoPS; *N* = 8) or coagulase-negative staphylococci (CoNS; *N* = 7), causing persistent mastitis (after several failed treatments) vs. infection-free (i.e., healthy [H]; *N* = 8) dairy cows. The expressions of the acute phase protein genes serum amyloid A3 (*SAA3*), haptoglobin (*HP*), ceruloplasmin (*CP*) genes in the tissues most exposed to pathogens— mammary gland cistern lining epithelial cells (CLECs) and mammary epithelial cells (MECs)—were analyzed.

**Results:**

We found constitutive and extrahepatic expressions of the studied genes in both tissue types. *HP* expression in the MECs of the CoPS-infected group was higher than in the H group (*p* ≤ 0.05). Moreover, higher *SAA3* expression in the CoPS and CoNS groups than in the H group (*p* = 0.06 and 0.08, respectively) was found. No differences between *SAA3* and *HP* in CLECs were revealed, regardless of the pathogen type. However, higher expression of *CP* (p ≤ 0.05) in the CoPS group than in the H group was noted.

**Conclusions:**

The expressions of selected acute phase proteins were similar between CLECs and MECs, which means that CLECs are not only a mechanical barrier but are also responsible for the biological immune response. Our findings agree with the results of other authors describing the immunological response of MECs during chronic mastitis, but the results for CLECs are novel.

## Background

Over the course of many years, breeding programs in the dairy industry have selected mainly for the traits of high milk yield and quality. This selection has resulted in highly productive dairy cattle; however, these animals are prone to many infections, especially those related to the mammary gland. Bovine mastitis is the most common disease in dairy cattle worldwide and is the costliest disease for the dairy industry [1]. Very complex processes occur during mastitis. Briefly, the disease is present when microbes overcome anatomical barriers, enter the udder, and activate cellular and soluble factors within the mammary gland. Occasionally, mastitis symptoms may occur in response to chemical, mechanical, or thermal trauma to the udder [2]. Moreover, toxins released by some bacteria damage the milk-secreting tissue and milk ducts, resulting in reduced milk yield and quality [3]. This damage can even lead to animals becoming unable to produce milk, which in turn results in animal culling [4].

Mastitis-causing staphylococci are divided into two groups: coagulase-positive staphylococci (CoPS; e.g., *Staphylococcus aureus*), which are considered major pathogens, or coagulase-negative staphylococci (CoNS; e.g., *S. xylosus, S. chromogenes*, or *S. warnei*), which until recently, have been considered less pathogenic (i.e., merely minor pathogens) [5]. However, CoNS have been identified as the predominant bacterial species isolated from ruminants with mastitis [6], and production of staphylococcal endotoxins similar to pathogenic *S. aureus* has been confirmed in many CoNS, e.g., *S. hemolitycus, S. epidermidis, S. chromogenes*, *S. warnei*, or *S. xylosus* [7].

The major role of lymphocytes, macrophages, neutrophils, and natural killer (NK) cells in response to mastitis has been well recognized. Mammary epithelial cells (MECs), which form the secretory tissue, also play a very well-known immunological role during udder inflammation. MECs are responsible for secreting a number of factors related to the host’s defense against pathogen invasion in ruminants (e.g., lactoferrin and antimicrobial peptides) [8, 9].

However, the udder’s first line of defense against pathogens is the lining epithelial cells, which line the whole teat canal and gland cistern. Rich in keratin, these cells are also located around the teat canal end and form the keratin plug that protects the teat canal entrance from pathogen intrusion; however, up to half an hour after milking, the canal stays open. The cells of this lining epithelial tissue are tightly connected, building a strong mechanical barrier that prevents pathogens from passing through. Yet, despite their importance, there is still very limited information about cisternal lining epithelial cells (CLECs) as a biological barrier. Suppression of a bacterial infection often depends on a quick immune response, mostly by the innate immune system, which occurs within hours after pathogen entrance [10]. Thus, if the CLECs release antimicrobial agents, such as acute phase proteins (APPs), their secretion at the initial step of the infection probably helps protect the udder against pathogens.

APPs are one of the organism’s first lines of defense against infection during the systemic reaction to inflammation. They belong to a heterogeneous group of proteins in terms of their structure, function, and mode of action, and are produced mainly in the liver. Moreover, their properties can differ significantly: some are anti-inflammatory, while others are pro-inflammatory. The concentration of these proteins is substantially altered during inflammation, trauma, or infection (i.e., acute phase reaction) as a result of complement system activation or after the release of various pro-inflammatory mediators [11]. Human APPs are the most recognized of these proteins, and they can be divided, in general, into two groups: 1) positive APPs, whose concentration increases after trauma, e.g., serum amyloid A (SAA), haptoglobin (HP), ceruloplasmin (CP), fibrinogen (FB), C-reactive protein (CRP), lipopolysaccharide binding protein (LBP), ferritin (FT), or lactoferrin (LF), and 2) negative APPs, whose reaction is opposite during acute phase reaction, e.g., albumins, transferrin (TF), or transthyretin (TTR). However, for different animal species, various other proteins have been recognized as APPs [12]. As of now, the following APPs have been recognized for cattle: SAA, HP, CP, FB, CRP, LBP, FT, LF, bovine cluster of differentiation 14 (CD14), and calcitonin gene-related peptide (CGRP) during different diseases, such as mammary gland infections, uterine infections, lameness, or fatty liver syndrome [12]. SAA and HP are the most valuable biomarkers of diseases, especially during mastitis [13]. CP is very well known as an inflammatory indicator in cattle, as it protects tissues from iron-mediated free radical injury [14]. It has been assessed as a marker of animal welfare and health. Moreover, researchers have also recognized its role during mastitis [12].

The aim of the study was to determine the expressions of *SAA3*, *HP*, and *CP* genes in MECs and CLECs during chronic subclinical mastitis caused by CoPS and CoNS vs. bacteria-free udder samples to compare the immune response of both tissues to staphylococcal infection.

## Results

**Fig. 1:**
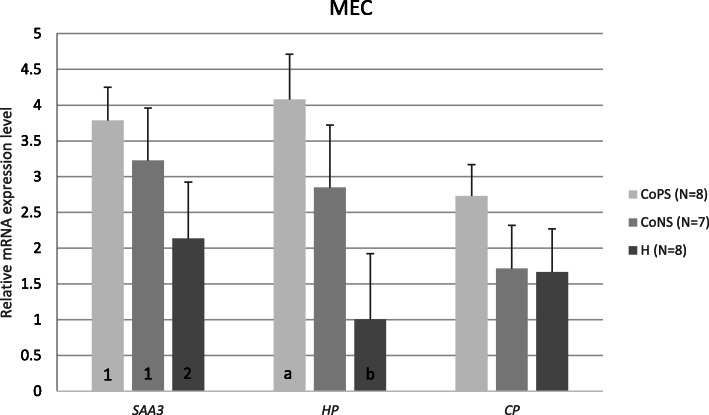
Means and their standard errors of mRNA levels of *SAA3*, *HP*, and *CP* genes in mammary gland epithelial cells (MEC) depending on the mammary gland health status: CoPS (*N* = 8), CoNS (*N* = 7), or H (*N* = 8). *SAA3* – serum amyloid A3 gene, *HP* – haptoglobin gene, *CP* – ceruloplasmin gene, CoPS – coagulase-positive staphylococci, CoNS – coagulase-negative staphylococci, H – samples free from bacteria. The value within the same gene with different letters differs significantly: a, b – at *p* ≤ 0.05. The value within the same gene with different numbers differs at the trend level: 1,2 – at *p* < 0.1

In this study, we found the constitutive expression of the studied genes within the analyzed tissues. Transcripts for all of the studied genes were found even in the infection-free samples of both types of tissues. However, *HP* expression in the MEC samples infected with CoPS was approximately four times higher than that of the H group (*p* ≤ 0.05). Moreover, we found a ~ 1.8-fold higher expression of *SAA3* in CoPS and a ~ 1.5-fold higher expression in CoNS than in the H group (*p* < 0.06 and 0.08, respectively). No differences in *CP* expression in MECs were found, regardless of the animal’s health status (Fig. 1).

**Fig. 2:**
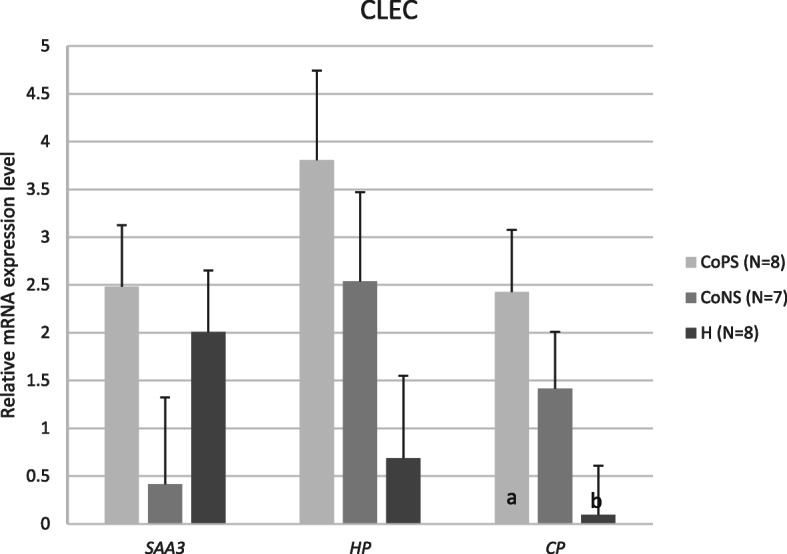
Means and their standard errors of mRNA levels of *SAA3*, *HP*, and *CP* genes in cistern lining epithelial cells, depending on the mammary gland health status: CoPS (*N* = 8), CoNS (*N* = 7), or H (*N* = 8). *SAA3* – serum amyloid A3 gene, *HP* – haptoglobin gene, *CP* – ceruloplasmin gene, CoPS – coagulase positive staphylococci, CoNS – coagulase-negative staphylococci, H – samples free from bacteria. The value within the same gene with different letters differs significantly: a, b – at p ≤ 0.05

Furthermore, no differences between *SAA3* and *HP* expression in CLECs were revealed, regardless of the pathogen type. However, we found a higher expression of *CP* (p ≤ 0.05) in the CoPS-infected samples than in the H samples (~ 25 times higher) (Fig. 2).

In the comparison of the selected APPs’ expression levels between CLECs and MECs with different pathogen types, no differences between *SAA3, HP,* and *CP* expression were found.

However, strong positive correlations between the mRNA levels of the *CP* and *SAA3* (*p* ≤ 0.01) and *CP* and *HP* (p ≤ 0.01) genes in CLECs were found, while no correlation between the transcript levels of the *SAA3* and *HP* genes was stated (Table 1). Similar observations were made in MECs, such as positive correlations between *SAA3* and *CP* gene mRNA levels (*p* ≤ 0.05) and between *CP* and *HP* gene expression levels (p ≤ 0.01), with the addition of a positive correlation between *SAA3* and *HP* expression levels (p ≤ 0.01). Moreover, an analysis of the selected APP gene expression patterns revealed a negative correlation for *CP* and *HP* between both tissues (p ≤ 0.05).

**Table 1:** Correlations between the target genes’ expression (*SAA3* – serum amyloid A3; *CP* – ceruloplasmin; *HP* haptoglobin, *CLECs* cisternal lining epithelial cells, *MECs* mammary epithelial cells, *NS* nonsignificant). The Pearson correlation coefficient and value of probability *p* (*p-value*) are shown in the table (*p ≤ 0.05; **p ≤ 0.01)

	*CP* CLECs	*HP* CLECs	*SAA3* MECs	*CP* MECs	*HP* MECs
*SAA3 P*CLECs	0.76**	NS	NS	NS	NS
*CP* CLECs		0.55**	NS	NS	NS
*HP* CLECs			NS	−0.45*	NS
*SAA3* MECs				0.46*	0.75**
*CP* MECs					0.76**

## Discussion

The higher expression of the *HP* gene in MECs infected with CoPS compared to that of the H group (p ≤ 0.05) may suggest that HP plays an important role as a major APP in cattle during chronic mastitis, which is consistent with results obtained by another research team [13]. The main role of HP is the binding of free hemoglobin. Iron-utilizing pathogens have developed many mechanisms for extracting iron from free hemoglobin. The binding of hemoglobin protects iron ions from being used by harmful bacteria. Moreover, free hemoglobin is highly toxic to tissues due to the lipophilic character of heme (i.e., it interacts with the cellular lipid bilayer), and the iron present in heme facilitates the generation of reactive oxygen species [15]. Our research illustrates the extrahepatic nature of HP synthesis with MECs—a finding that is in line with other scientific reports [16–18].

The higher expression of the *SAA3* gene in samples infected with CoPS and CoNS compared to the control group may imply a crucial role for SAA, together with HP, during chronic mammary gland inflammation. Although the main roles of SAA3 are the binding and transportation of lipoproteins, it also plays an important role in the immune system, e.g., SAA3 activates neutrophils and macrophages and their migration, stimulates T cell adhesion, participates in monocyte chemotaxis, and also facilitates lymphocyte and endothelial cell proliferation [19]. The elevated level of *SAA3* and *HP* mRNA transcripts in MECs during mastitis found in our study is in accordance with a study conducted by Eckersal et al. [20]. That team found increased *HP* and *SAA3* transcript levels in mammary gland tissue at the trend level, specifically in MECs as well as in CLECs, after an experimental infusion of *S. aureus* (the animals were kept for ~ 30 days and then euthanized 48 h after the last bacterial infusion). Furthermore, in their immunocytochemical study, the researchers found a higher concentration of SAA3 within the infected tissues.

We have shown the presence of *CP* mRNA transcripts within both infected and bacteria-free samples, which implies the constitutive expression of this gene. However, in CLECs, a higher expression level within tissue infected with CoPS compared to H was found (*p* ≤ 0.05), while no differences in MECs were observed. The presence of mRNA transcripts of this gene in both analyzed tissues indicates its extrahepatic expression; however, although several studies have reported *CP* expression in mammary gland secretory tissue, the liver remains the main source of this APP [19]. The higher level of *CP* transcription in the CLECs of the CoPS vs. the H samples may suggest an increased presence of reactive oxygen species during this type of infection [21]. The elevated level of *HP* and *CP* gene expression within the tested tissues may be related to their main role, which is the binding of free iron ions. As part of the immune response, these proteins keep iron ions tightly bound, which inhibits pathogens from utilizing the iron, thus protecting the organism. This function prevents bacteria from propagating, as they need iron to grow in the udder [12]. An elevated level of these proteins during chronic infections could be related to their protective role against tissue damage caused by pathogens. Moreover, CP exhibits oxidase activity, e.g., catechol or amine oxidase activity, toward different substituted organic compounds. In addition, this protein may act as a scavenger of reactive oxygen species, such as singlet, superoxide, and hydroxyl radicals [21].

In our study, all tested samples from the CoPS and CoNS groups were obtained from dairy cattle suffering from naturally infected mammary glands after several failed antimicrobials therapies. The animals from the experimental groups were culled because of recurrent, chronic, and incurable udder inflammation. Therefore, the obtained results show the importance of SAA3 and HP during persistent infection in MECs and of CP in CLECs. During acute inflammation, the concentration of APPs rises up to 100-fold in the first 48 h, while during chronic inflammation, which occurs usually after acute inflammation, the concentration of APPs is only 10-fold higher than in healthy tissues as a result of its decreasing concentration after the acute phase [22]. These findings are consistent with our results however, the 24-fold higher expression of the *CP* gene in CLECs infected with CoPS vs. those of the H group may instead indicate that the acute response of CLECs to staphylococcal infection is ongoing, despite the persistent form of the disease.

Other research teams have obtained results similar to ours, but considering the unique character of our analysis, it was challenging to find research data on mRNA transcript levels to compare with ours. However, some study have been conducted at the protein level. Moreover, it was difficult to find any other studies with the same animal model as the one we used. Horadagoda et al. [23] observed elevated levels of SAA3 and HP proteins in the blood serum of cows diagnosed with different types of chronic, as well as acute, inflammation (including mastitis), showing higher SAA3 and HP concentrations during the acute state compared to the chronic one. However, some of our results differ from the literature because of differences in the research model. In general, most knowledge about APPs has been gained from a laboratory-induced model, and much of the research has been conducted in a short period of time, e.g., 24–72 h after bacterial challenge.

MECs are very well known for their biological role during mammary gland inflammation. This secretory tissue is not only responsible for milk secretion but is also, upon interaction with invading bacteria, able to produce different pro-inflammatory or anti-inflammatory mediators such as cytokines, chemokines, APPs, as well as antimicrobial peptides and proteins (β-defensins, cathelicidins, and LF) [24, 25]. Moreover, these cells may be involved in recruiting neutrophils and lymphocytes to milk [26]. In contrast, CLECs, present in teats, a milk cistern, and ducts, have always been considered to form a strong mechanical barrier against bacterial invasion to the gland due to the strong and tight connections between cells. It is the very first tissue that comes into contact with pathogens invading the udder and is the most exposed to contact with microorganisms. Knowledge of the role of CLECs in the innate immune system is very limited. A number of studies have revealed that other cell types are involved in the immune activity within the udder, such as MECs [27, 28] and probably CLECs, as it was shown in our study.

Our research shows the elevated expression of *SAA3* (*p* < 0.1) in MECs for CoPS and CoNS groups compared to the H group, which may suggest the comparable role of this APP regardless of pathogen type during chronic infection caused by Gram-positive bacteria. This assumption may prove that it belongs to the main APPs in dairy cattle. However, our study revealed high individual variations within the groups, thus further research with larger experimental groups is needed to fully elucidate this thesis.

CoPS have always been considered major pathogens. The pathogenicity of this group of bacteria is related to the virulence factors of the causative agent, and eventually, resistance to commonly used antibiotics develops [29]. In contrast, until recently, CoNS have been regarded as environmental microorganisms that are harmless until specific conditions occur (e.g., a temporary weakening of the immune system). Formerly, mastitis caused by CoNS was often left untreated because the spontaneous cure rate of this condition is considered high (16–70%) [30]. However, recently scientists have started to recognize CoNS as a potential threat to animal health. Infection caused by CoNS could be persistent during lactation, similar to mastitis caused by CoPS [31]. It should be stressed that CoNS have been found to be the most common udder infection-causing bacterial pathogen isolated from milk samples, thus they could be described as emerging pathogens (despite their initial classification) [6]. Moreover, CoNS infection is usually connected with a mild increase in somatic cell count, while CoPS bacteria usually cause a much higher increase. However, as it was mentioned above, an organism with strong immunity is able to cope with CoNS by itself, while it is usually not able to fight CoPS bacteria, especially *S. aureus* [1, 6].

In summary, we observed a higher expression level of *CP* within the CLECs of the CoPS samples, as well as a higher level of *HP* mRNA transcription within the MECs of the CoPS samples compared to those of the H group (*p* ≤ 0.05), which may suggest different infection and iron acquisition mechanisms in these two groups of pathogens. Almost all living organisms require iron for living, and staphylococci are no exception. There is a lack of easily accessible iron in vertebrate tissue due to the presence of high-affinity iron-binding proteins, such as TF and LT. However, some *S. aureus* isolates may exhibit hemolytic activity to obtain iron from heme. These strains grow well under iron-restricted conditions due to the ability of the strains to produce siderophores (high-affinity iron-binding molecules). Conversely, only a small number of CoNS isolates produce siderophores, and most grow poorly in an iron-limited environment [32]. Thus, during mastitis caused by staphylococci, there is an urgent need to protect iron from being “downloaded” by bacteria, and it is probably the reason for the elevated expression of the genes encoding iron-binding APPs in the CoPS group in our study.

We did not note any differences in immunological responses in the selected APPs’ transcript levels for MECs or CLECs. We found comparable expression of selected APP genes within both analyzed tissue types for the same pathogen. This finding may suggest that both tissues react similarly during mastitis. It may also imply that during chronic mammary gland inflammation, when bacteria are present for a longer time in the whole udder, the tissues produce different molecules, such as APPs, to combat infection and reduce pathogenic propagation; however, these molecules are produced at a lower level than during acute inflammation to ensure that the host’s cells are not permanently harmed. These findings are in line with different publications describing the immunological response of MECs during mammary gland inflammation [10, 17, 24, 25], but the results for CLECs obtained in this study have not been described before.

The strong positive correlations between the transcripts of the *CP* and *SAA3* genes and between those of the *CP* and *HP* genes in CLECs imply the combined action of their coded proteins against infection within these cells during chronic mastitis. Similarly, the correlations between the mRNA levels of the *CP* and *SAA3*, the *CP* and *HP*, and the *SAA3* and *HP* genes within MECs may suggest the combined action of the genes’ protein products to combat persistent inflammation, especially since the proteins encoded by these genes participate in iron metabolism. However, the negative correlation found between *CP* and *HP* gene expressions in both studied tissues may suggest their different iron-binding properties during bacterial infection in these two tissues.

It should be noted that this study was conducted on a relatively small group of animals—23 cows, with two types of tissue per cow. However, cows were homologous in term of breed, animal keeping conditions, and stage of lactation. During the study, only the two different types of staphylococci were present—no *E. coli* nor streptococci were detected. The cows were naturally infected with staphylococci and culled because of recurrent incurable mastitis. Thus, the time from the infection was different for each cow, and the animals had undergone a different number of therapies. Moreover, the study was especially focused on the chronic form of the inflammation, thus cows with clinical mastitis were excluded from the experimental groups to eliminate acute responses to infection. However, all cows were assigned to their experimental groups approximately 1 month after their last therapy, while their somatic cell count in milk remained elevated: the median value was 2.44 × 10^6^/mL for CoPS and 4.61 × 10^5^/mL for CoNS.

## Conclusions

In summary, in this study, the constitutive and extrahepatic expression of the studied APPs was reported*.* We also found that the transcript levels of selected APPs were similar between CLECs and MECs, which means that CLECs not only act as mechanical barriers but also play important biological roles during chronic mastitis. Thus, APPs secreted by those cells may play a crucial role in the protection of the udder against pathogens.

## Methods

### Animals and tissue samples

The study was conducted on 23 Polish Holstein-Friesian dairy cows of the Black-and-White variety, which were maintained on the Experimental Farm at the Institute of Genetics and Animal Breeding in Jastrzębiec, near Warsaw, Poland. The cows were between their first and fourth lactation, and those from the experimental groups had naturally occurring chronic mastitis. The conditions in which the cows were kept were previously described by Kościuczuk et al. [25]. The herd participates in the milk recording system, and complete information was available on the milk parameters of the animals, including somatic cell count, as well as on the number of treatments of each cow over the course of her life. All animals were under constant veterinary supervision. According to herd management, the animals were culled due to recurrent udder problems, diagnosed by a veterinarian based on clinical or subclinical signs of mastitis (flakes or clots in milk, somatic cell count) and milk microbiological examination, and after several failed therapies with antimicrobials (elevated somatic cell count during the entire last lactation despite therapies). The samples for the experimental groups were derived from these animals; however, those with clinical signs of mastitis (acute inflammation) were excluded from the study. Eight cows were culled due to reproduction problems (no pathogenic bacteria in milk and a somatic cell count below 15 × 10^4^ cells/mL during the whole lactation). The samples derived from these animals served as the control group. All cows were culled at the end of lactation (approximately 280 days, SD = 25). They were slaughtered at the registered certified slaughterhouse under constant monitoring by authorities at least 1 month after their last antimicrobial administration during a two-stage process for the non-ritual slaughter: electrical stunning to render an animal unconscious and then exsanguination was done. MEC and CLEC samples were obtained immediately after the animals were slaughtered. CLEC samples were taken from the bottom part of the gland cistern, in close proximity to the teat cistern. The MEC samples were collected from deep inside the secretory tissue of the gland. One sample of each tissue (from only one udder quarter from only one cow) was used in the present study. The samples were immediately washed in ice-cold phosphate-buffered saline (PBS; pH 7.2) to remove any blood and milk contamination and then were instantly frozen in liquid nitrogen. Tissue samples were stored at − 80 °C prior to subsequent analysis. The samples were derived from all cows culled from the herd in 2010–2013, and all eligible samples were included in the analysis. The assessors were blinded to any stages of the methodological process to limit the occurrence of conscious and unconscious bias in the conduct of trials and interpretation of outcomes.

### Methods

#### Microbiological analysis of milk

The samples of ‘first milk’ were collected aseptically from each quarter of the udder 2 days before slaughter and were tested for the presence of bacteria. To identify microorganisms, 100 μl of the milk sample was inoculated on Columbia agar supplemented with 5% sheep blood (BioMaxima, Lublin, Poland). The plates were incubated at 37 °C for 24 h. All isolates were assessed for phenotype, the morphology of the colony, and biochemical properties (API – analytical profile index; bioMérieux, Craponne, France). Coagulase production ability was tested via the rabbit plasma tube test. Furthermore, a SLIDEX StaphKit (bioMérieux) was used for *S. aureus* identification.

#### RNA isolation

RNA was isolated from tissue samples with the commercially available RNeasy Mini Kit (Qiagen, Hilden, Germany) according to the manufacturer’s protocol. Qualitative and quantitative analyses of RNA were performed using a NanoDrop1000 spectrophotometer (Spectro-Lab, Warsaw, Poland) and a 2100 Bioanalyzer (Agilent Technologies, Santa Clara, USA). Samples with an RNA integrity number value greater than seven were selected for further analysis. Reverse transcription reactions were performed using the Transcriptor First Strand cDNA Synthesis Kit (Roche, Meylan, France) following manufacturer’s protocol.

#### Gene expression analysis

Expression levels of the *SAA3*, *HP*, and *CP* genes were analyzed using an RT-qPCR LightCycler 480 system (Roche, Meyla, France) on 96-well plates with the SYBR Green technique, according to the manufacturer’s protocol. Primer sequences designed by Whelehan et al. [33] were used for qPCR analysis. Information on the primer sequences, amplicon sizes, annealing temperatures, and GenBank accession numbers is shown in Table 2. Three types of negative controls were included: no template addition, no reverse transcriptase addition, and no polymerase addition. The presence of the product of interest was confirmed by electrophoresis in 2% agarose gel (G:BOX visualization system, Syngene, Cambridge, UK). Glyceraldehyde 3-phosphate dehydrogenase (*GAPDH*) was used as a reference. The process of housekeeping gene (HKG) selection in MECs has been described by Kościuczuk et al. [25], and the *GAPDH* was one of the genes with an M below 0.5. The present study was conducted on the samples derived from the same animals as in the above-mentioned study.

**Table 2:** Gene names and abbreviations, primer sequences, amplicon sizes, annealing temperatures, and accession numbers

Gene name	Gene abbr.	Primer sequence	Amplicon size [bp]	Annealing temp [°C]	GenBank accession number
Serum amyloid A3	*SAA3*	CTCAAGGAAGCTGGTCAAGG CTTCGAATCCTCCCGTACCT	240	58	NM_181016
Ceruloplasmin	*CP*	TTCATGCACATGGAATGACTT TAAAGGCCCAATGAGTCCTG	236	58	NM_001256556.1
Haptoglobin	*HP*	TGGTCTCCCAGCATAACCT AGGGTGGAGAACCACCTTCT	185	58	NM_001040470

The C_T_ values obtained from qPCR results were calculated according to a modified version of Pfaffl’s formula [34]:

$$ ratio=\frac{{\left({E}_{target}\right)}^{{\varDelta CP}_{target}^{\left( mean- sample\right)}}}{{\left(E| ref\right)}^{{\varDelta CP}_{ref}^{\left( mean- sample\right)}}} $$

where:

ratio – the relative proportion of the tested gene expression (target) to the reference gene (ref) expression.

E – qPCR efficiency for the target gene (E_target_) or for the reference gene (E_ref_).

CP – crossing-point qPCR value: the cycle at which the fluorescence rises above the background fluorescence (defined threshold).

∆CP_target_ – CP deviation of mean expression minus the expression of the targeted gene in a sample.

∆CP_ref_ – CP deviation of mean expression minus the expression of the reference gene in a sample.

mean – the average arithmetic value of the CP from all reactions for the studied gene (in the numerator) or for the reference gene (in the denominator).

sample – each CP value for the studied gene (in the numerator) or for the reference gene (in the denominator) for each sample.

### Statistical analysis

All collected samples were divided by tissue type (MECs or CLECs) and antimicrobial agent (CoPS (*N* = 8) or CoNS (*N* = 7) or bacteria-free samples (H; *N* = 8). Altogether, 46 tissue samples were obtained: 23 samples per tissue type.

To search for differences in gene expression levels, analyses of variance were performed using the ANOVA procedure with post-hoc Tukey-Kramer test (SAS/STAT 2002–2012, ver. 9.4), taking into account the fixed effect of the interaction between the microbiological status of the milk (CoPS-infected, CoNS-infected, H) and the tissue type, and error as random.

The normality of the distribution of all traits was checked using a Univariate procedure (SAS/STAT, 2002–2012, ver. 9.4), and values for the expression of genes at the mRNA level were transformed into a natural logarithmic scale.

The authors chose the following cut-off points for significance: the values differ significantly at *p* ≤ 0.01 (indicated as A, B); the values differ significantly at *p* ≤ 0.05 (indicated as a, b); the values differ at the trend level at 0.05 < *p* < 0.1 (indicated as 1, 2); and the values do not differ significantly at *p* ≥ 0.1.

The final statistical model did not include the lactation number because in a prior analysis, lactation number did not influence the expression of the selected genes.

## Data Availability

The datasets analyzed during the current study are available from the corresponding author upon request.

## Abbreviations

API: Analytical profile index
APP: Acute phase protein;
CD14: Cluster of differentiation 14
CGRP: Calcitonin gene-related peptide
CLEC: Cistern lining epithelial cell
CoNS: Coagulase-negative staphylococci
CoPS: Coagulase-positive staphylococci
CP: Ceruloplasmin
CRP: C-reactive protein
FB: Fibrinogen
FT: Ferritin
GAPDH: Glyceraldehyde 3-phosphate dehydrogenase
HP: Haptoglobin
LBP: Lipopolysaccharide binding protein
LF: Lactoferrin
MEC: Mammary epithelial cell
PBS: Phosphate-buffered saline
SAA3: Serum amyloid A3
TF: Transferrin
TTR: Transthyretin
